# Detection of Soluble Amyloid-**β** Oligomers and Insoluble High-Molecular-Weight Particles in CSF: Development of Methods with Potential for Diagnosis and Therapy Monitoring of Alzheimer's Disease

**DOI:** 10.4061/2011/151645

**Published:** 2011-11-02

**Authors:** Susanne Aileen Funke

**Affiliations:** Forschungszentrum Jülich, ICS-6, Wilhelm-Johnen-Str., 52425 Jülich, Germany

## Abstract

The diagnosis of probable Alzheimer's disease (AD) can be established premortem based on clinical criteria like neuropsychological tests. Post mortem, specific neuropathological changes like amyloid plaques define AD. However, the standard criteria based on medical history and mental status examinations do not take into account the long preclinical features of the disease, and a biomarker for improved diagnosis of AD is urgently needed. In a large number of studies, amyloid-**β** (A**β**) monomer concentrations in CSF of AD patients are consistently and significantly reduced when compared to healthy controls. Therefore, monomeric A**β** in CSF was suggested to be a helpful biomarker for the diagnosis of preclinical AD. However, not the monomeric form, but A**β** oligomers have been shown to be the toxic species in AD pathology, and their quantification and characterization could facilitate AD diagnosis and therapy monitoring. Here, we review the current status of assay development to reliably and routinely detect A**β** oligomers and high-molecular-weight particles in CSF.

## 1. Introduction

Alzheimer's disease (AD) is a detrimental neurodegenerative disorder and the most common cause of dementia. AD results in memory loss and behavioral problems, leading to disastrous impact on the patient's life and on that of the patient's relatives. It was estimated that approximately 27 million people are affected worldwide. As aging is one of the main risk factors for AD and people grow steadily older, the number of afflicted people is expected to quadruble by 2050 [1].

The amyloid-*β* (A*β*) peptide has been identified to play a major role in the pathology of AD. The 4 kDa peptide, consisting of 39 to 42 amino acid residues, is derived from the proteolysis of the amyloid precursor protein (APP) by two different proteases, *β*- and *γ*-secretase [2–4]. A*β* is the main component of amyloid plaques associated with AD [5]. The amyloid cascade hypothesis states that A*β* aggregation followed by plaque formation is a central event in AD [6]. Today, it is well known that diffusable and soluble A*β* oligomeric species are the main toxic species in AD. A*β* oligomers have been shown to impair long-term potentiation (LTP), decrease the density of dendritic spines in hippocampal brain slices *in vitro* and impair memory *in vivo*. Fibrillar A*β* deposited in plaques was shown to exhibit comparably low toxic effects, and the plaque load in the brain does not correlate well with the symptomatic disease progress [7–10]. Today, the diagnosis of definite AD requires clinical diagnosis, based on the observation of clinical symptoms, and postmortem detection of amyloid plaques and neurofibrillary tangles, the latter composed of aggregated tau protein, in the brain tissue of the deceased patient. The diagnosis “probable AD” can be established with 50% to 90% certainty dependent on clinical criteria, neuropsychological testing, and laboratory tests [11–13]. The first molecular events leading to AD, like A*β* oligomerization and plaque deposition as well as tau pathology, are supposed to appear 10 to 20 years before the symptoms become apparent [11–16]. Therefore, new criteria for diagnostic research have been proposed with the aim to incorporate supportive biomarker information, for example, CSF A*β* and tau monomer concentration, glucose and amyloid imaging using positron emission tomography (PET), and magnetic resonance spectroscopy (MRS) for the detection of brain atrophy to allow a more sensitive and specific diagnosis of AD in preclinical stages, that is, when symptoms are not yet apparent [17, 18].

Biomarkers that are directly or indirectly related to the pathophysiological changes of AD might be auxiliary to diagnose AD differentially in preclinical stages. Early diagnosis will aid treatment decisions substantially, as currently the majority of scientists agree that AD treatment will be most effective in early stages of the disease [19]. At present, only symptomatic treatment of AD is available, but several compounds are currently being developed, most of them aiming at A*β*, for example, secretase inhibitors, immunotherapy and A*β* aggregation inhibitors [19–21]. More than 10 compounds are currently in clinical phase III trials, and several more in phase I or II. Referring to PubMed, hundreds of compounds are in the preclinical state. Furthermore, biomarkers will be needed to select and characterize the patients to be enrolled in clinical studies as well as to monitor the effects of the drug candidates [22].

A variety of studies have shown that reduced levels of A*β*1-42 monomers, in most cases determined by ELISA or related methods, implicate a high predictive value for the identification of prodromal AD in cases of mild cognitive impairment (MCI). CSF concentrations of monomeric A*β*1-42 are reduced by 30 to 50 percent in AD patients compared to age-matched, nondemented controls as confirmed in many independent studies, with both sensitivity and specificity exceeding 80 to 90% in most of them. There is evidence suggesting that combined biochemical analysis of tau and phosphorylated tau, which both are increased in CSF of AD patients in comparison to controls, and A*β* measurements in CSF can improve the diagnostic value as it can even predict AD with sensitivity and specificity values of 80 to 90% [23]. A variety of biomarker studies have also been performed in plasma, but the results are rather inconsistent, and at present the convenience to detect A*β*1-42 as a biomarker in plasma has not been proven yet (reviewed in [24, 25]). 

**Figure 1:**
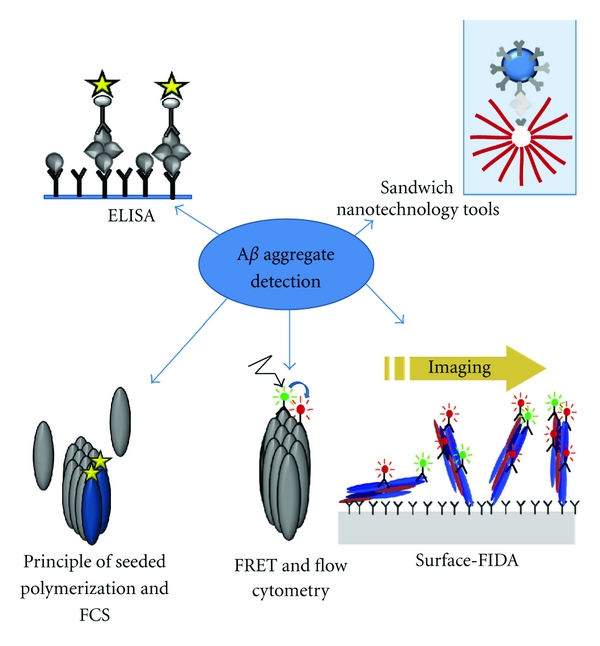
Summary of method types used for the detection of A*β* oligomers in CSF. Sandwich-ELISA methods provide oligomer specificity because the same antibody is applied as capture and for detection. Alternatively, oligomer-specific antibodies can be used in the detection process. In sandwich nanotechnology tools, two specific antibodies frame A*β* oligomers. The signal of detection is amplified, for example, by conjugated gold nanoparticles with hundreds of DNA barcodes attached in biobarcode assays. The DNA-magnet-sandwich complexes are extracted from the sample using a magnet. Subsequently, the total number of DNA barcodes is determined. In assays based on seeded polymerization, preexisting multimeric A*β* particles in body fluids are spiked by adding labeled A*β* peptides to the sample. Detection is performed via fluorescence correlation spectroscopy (FCS). Fluorescence resonance energy transfer (FRET) is a mechanism describing energy transfer between two chromophores in spatial proximity, which is given between two fluorophores attached to the same A*β* oligomer. FRET signals are detected by flow cytometry. Surface-FIDA is based on a laser focus scanning the surface of a specially prepared glass chip. Either a fluorescence correlation spectroscopy (FCS) device or a laser scanning microscope (LSM) can be used. A*β* aggregates or oligomers are concentrated in a two-dimensional surface by immobilizing them on a glass slide using A*β* capture antibodies. The aggregates are detected by adding at least two fluorescence-labeled anti-A*β* antibodies. At least, two laser beams are focused on the surface of the glass chip, and the fluorescence light which is emitted by the fluorescence antibodies is detected in a confocal way, enabling single aggregate detection. A quantitative value for specific colocalized fluorescence pixels is yielded by the summation of all cross-correlated pixels in the colocalization area above a threshold (cutoff) intensity value. Only double-labeled events are considered for the analysis.

However, the monomeric form of A*β* is not the main responsible isoform for neurotoxicity and neurodegeneration. A*β* oligomers have been shown to play a fundamental neurotoxic role in AD pathology, and the ability to quantify and qualify them as well as insoluble high-molecular-weight (HMW) aggregates could not only enhance AD diagnosis, but also help to investigate the contribution of A*β* aggregates to AD pathology. The development of techniques for the reliable detection of A*β* aggregates, however, is technically challenging due to the heterogeneous and unstable nature of such particles, which steadily interconvert into each other, and their low abundance. Here, we review the current status of assay development to reliably and routinely detect A*β* oligomers and HMW particles in CSF. We especially focus on the development of methods with single A*β* aggregate sensitivity. Methods which were successfully developed but not yet applied to human body fluids were not included. All types of methods reviewed here are summarized in Figure 1. In this review, the term “aggregates” describes all kinds of non-monomeric A*β* conformers including oligomers, protofibrils and fibrils. A*β* oligomers are defined as nonfibrillar, soluble low-molecular-weight conformers. The term HMW particles describes unsoluble A*β* conformers. Phrases describing special A*β* species are used as by the authors of the reviewed methods.

## 2. Methods for the Detection of A***β*** Aggregates in CSF, Resulting in One Summarized Signal

Starting in 2005, a variety of articles were published that describe techniques for the detection of A*β* aggregates in body fluids. All methods are based on A*β* caption using specific antibodies, and they result in the summarized quantity of A*β* aggregates as readout, either via nanotechnology or ELISA-based tools. The detection of A*β* monomers is excluded either by oligomer specific antibodies or application of the same A*β*-binding antibody twice in the system, for example, as capture as well as detection antibody. Because the binding epitope in monomers will already be occupied by the capture antibody, dimers are supposed to be the smallest detectable unit. The latter methods will also detect HMW A*β* particles if the samples were not centrifuged before testing.

In 2005, two articles reporting on extremely sensitive nanotechnology-based assays for the detection of A*β*-derived diffusible ligands (ADDLs) were published. Haes et al. combined ADDL-specific antibodies with localized surface plasmon resonance (LSPR) spectroscopy and investigated the CSF of one AD patient and one control. Georganopoulou et al. applied-ADDL specific antibodies to develop an ultrasensitive barcode assay for specific detection of ADDLs in CSF. CSF samples of 15 AD patients and 15 controls were investigated. In both preliminary studies, the AD patient samples were shown to exhibit higher ADDL concentrations compared to the controls [26, 27]. After initial publications, no follow-up articles were published of both ADDL-specific assays. That might be due to technical difficulties of the technologies as the reported protocols contained several critical steps in sample preparation and measurement procedures, and suitability for application in high throughput or multicenter studies might be limited.

In 2010, Fukomoto et al. reported on a novel ELISA method for the specific detection of high-molecular-weight (HMW) A*β* oligomers. In the protocol, the same N-terminal binding A*β* antibody BAN50 was used for capturing the oligomers as well as to detect them. In CSF samples of 18 AD and 7 MCI cases, significantly higher signals for A*β* HMW oligomers were detected as in the 25 nondemented controls. Additionally, an inverse correlation of A*β* oligomer readout and MMSE scores could be described. The size of the detected oligomers was determined to be 40 to 200 kDa, mainly fractionating in size exclusion chromatography experiments at 45 to 90 kDa, representing mainly 10 to 20 mers. Monomers and lower-molecular-weight oligomer species were not detected [28]. Therefore, the authors provide a comparatively technically simple method to detect a special subset of A*β* conformers.

In 2010, Gao et al. developed peptides which bind A*β* aggregates and used them as bait for A*β* aggregates in CSF in the “misfolded protein assay” (MPA). After the aggregates were bound to aggregate-specific beads, A*β* was denatured and the concentration thereof was determined in A*β*1-40 and A*β*1-42 monomer-specific ELISAs. Interestingly, in CSF samples of 26 AD patients, higher amounts of A*β*1-40 aggregates could be detected than in the samples of 10 age-matched controls [29]. The nature of the detected aggregates remains unclear, as the aggregate-specific beads were described to bind higher-order aggregates as well as A*β* oligomers, and A*β* is denatured for concentration determination using conventional ELISA for A*β* monomers. This information, however, could be helpful for characterization of AD pathology and development of diagnostic approaches.

In general, the application of ELISA or related assay systems to analyze body fluids for their content of A*β* aggregates has several advantages. ELISA studies are easy to perform and technically simple, rendering them suitable for multicenter research. In case A*β* species-specific antibodies are employed and only one special A*β* conformer is detected, care has to be taken that the chosen conformer is really relevant for AD pathology and diagnosis. A variety A*β* oligomeric conformers, ranging from dimers to high-molecular-weight species, have been described *in vitro* and *in vivo*, being highly diverse with regard to structure and shape [7–9, 30–32]. The identity of the most relevant A*β* conformer is currently an active and controversial research topic and might be dependent of the stage of the disease. 

Up to date, a variety of conformer-specific A*β* antibodies were described, being specific either for A*β* oligomers, protofibrils or fibrils [33–37]. Only some of them were employed in biomarker studies for the detection of ADDLs in CSF [26, 27] or protofibrils in CSF and blood [38, 39]. To my knowledge, the oligomer-specific monoclonal antibodies A11 (Millipore), which recognizes all types of amyloid oligomers like, for example, prions, but not monomers and fibrils [36], and another A*β* oligomer-specific antibody (clone 4D8, Gentaur Molecular Products) are commercially available but were not used in any reported biomarker study yet.

Potential cross-reactivity of conformational specific antibodies for A*β* monomers has to be excluded carefully. Klaver et al. tested the specificity and sensitivity of an ELISA procedure in which the N-terminal binding A*β* antibody 6E10 was used as capture and for detection. It could be shown that in the described assay, A*β* monomers were detected at least to a certain extent [40]. Additionally, Sehlin et al. stated that positive results generated by A*β* oligomer ELISA assays could be caused by heterophilic antibodies, which are abundant in CSF and recognize immunoglobulins of other species. Heterophilic antibodies interfered in sandwich immunoassays by cross-binding capture and detection antibodies and caused false positive results [41]. Another drawback of ELISA studies for the detection of oligomers might be the underestimation of the number of molecules which was described by Stenh et al. [42].

One disadvantage of the methods described above is that one summarized signal for all particles under investigation is generated. In case conformer-specific antibodies are applied, structurally different A*β* oligomer species in the sample will remain undetected. In the next paragraph of this review, we will focus on methods based on single particle detection methodologies like fluorescence correlation spectroscopy and laser scanning microscopy. Methods with single particle sensitivity allow, per definition, the most sensitive detection of A*β* oligomers and HMW particles. In addition, in some of the methods, characterization of the single particles under investigation, for example, in respect of size, form, texture, and composition, is possible. At the end, detailed quantification and characterization of A*β* aggregates might lead to a better understanding of the contribution of A*β* oligomers and HMW particles to AD pathology.

## 3. Methods for Detection and Characterization of Single A***β*** Aggregates in CSF

To date, only very few methods for the detection of single A*β* aggregates in body fluids were described in the literature. One reason might be that technologies to detect at the single molecule level, like fluorescence correlation spectroscopy (FCS), laser scanning microscopy (LSM) with sensitive detectors and flow cytometry, are prone to fluorescence background or other artifacts and can generate false positive signals. Signals arising from A*β* oligomers, which have a comparably small size, have to be clearly distinguished from signals arising from monomers or from fluorescent background, and data analysis has to be adapted very carefully only to count the signals from A*β* conformers that contribute to AD pathology.

The first method for the detection of single A*β* aggregates in CSF was described by Pitschke et al. in 1998. The authors employed the process of seeded polymerization and detected A*β* aggregates in the CSF of AD patients using FCS. FCS means correlation analysis of the fluctuations of the fluorescence intensities of particles, which vary versus time due to Brownian motion in solution. Laser light is focused into a sample passing a dichroic mirror. When fluorescence-labeled particles cross the focal volume, they fluoresce and the emitted light reaches a very sensitive photomultiplier tube or avalanche photodiode detector. Light derived from out-of-focus areas is suppressed by a small aperture in front of the detectors. In typical FCS applications, the average number of fluorescent particles and their average diffusion time can be determined and concentration and size of the particles can be calculated. The fluorescence of particles to be investigated can be derived by themselves, by labels, or by addition of fluorescent ligands like antibodies. 

Pitschke and coworkers added labeled A*β* peptide, which was kept in SDS to avoid self-multimerization, to CSF samples of 15 AD patients and 19 controls. In contrast to regular FCS applications described above, the fluorescence intensity signal versus time was analyzed, and the frequency of high-intensity fluorescence peaks was calculated per minute. Samples from the controls produced a fluctuating, relatively low fluorescence intensity signal up to 20 minutes after addition of labeled A*β*. In the samples of the AD patients, high-intensity fluorescence bursts were detectable. In the latter, multimeric A*β* particles acted as seeds for rapid polymerization of the labeled A*β* monomers, and additional experiments attested that the major component of the particles was in deed A*β*. Smaller-intensity peaks detected in the control samples were derived from spontaneous multimerization of the fluorescent probes, but could be clearly distinguished from positive signals. The linearity of the assay was tested with synthetic A*β* and could be verified to be between 20 ng to 1000 ng in 20 *μ*L sample volume [43]. There is only limited information about the size of the aggregates which were originally present in the CSF before addition of fluorescence-labeled A*β* monomers. Only the fluorescence intensity of each single particle could give hints on aggregate size. This first article was the starting point for the development of methods for detection of A*β* oligomers or HMW particles in body fluids. 

In 2007, Henkel et al. refined the method described by Pitschke and colleagues and detected large A*β*1-42-binding particles (denoted LAPs) in the CSF of human AD patients and controls using a similar measurement principle as described above. The detection sensitivity of the self-made confocal assay-system was increased 20-fold in (indirect) comparison to the system described by Pitschke et al., employing a microchannel flow-through system and a sample velocity of approximately 0.72 mm/s. The analysis revealed that spiking CSF samples with fluorescent A*β* resulted in binding of the probe to particles preexisting in CSF, producing peaks of high fluorescence intensity. A*β* autoaggregates, already significantly reduced by filtering the fluorescence-labeled A*β*, were excluded from the analysis via definition of an intensity cut-off. Only very bright LAPs, three times as the threshold used by Pitschke et al., were included. LAPs were detected in samples of 8 AD patients and 6 patients with mixed-type AD as well as in controls (6 nondemented, 10 other neurodegenerative diseases) with high interindividual variation, but LAP concentration was not specific for AD. 

Next, the group used confocal microscopy to investigate the particles which acted as seeds in the samples in more detail. To detect the particles in the fluorescent images, A*β*1-42 labeled with Cy3 was added to the samples. Using an imaging analysis routine, area, shape, brightness, and texture of the particles were determined and the LAPs were grouped into four classes. A*β* autoaggregates could be clearly defined by low brightness, small size, and heterogeneous texture. LAP-1 aggregates were rarely found in CSF and resembled particles detected when labeled A*β* was added to synthetic A*β* seeds. LAP-2 aggregates seemed to contain protein-bound A*β* aggregates. Both LAP-3 and LAP-4 were bright particles, either ellipsoid (LAP-3) or round shaped (LAP-4). They resembled immune complexes that are observed in autoimmune diseases. LAPs-4 were virtually absent in all AD patients but present in approximately 40% of the control samples. This coherence was discussed as further evidence for a circulating IgG-based clearance system for soluble A*β* conformers [44]. In other studies, naturally occurring A*β* autoantibodies were detected to a higher extent in plasma samples of controls, in comparison to AD patients [45]. The method described by Henkel et al. revealed very interesting information about LAPs. In the images, however, the LAPs look comparably large (up to 2 *μ*M), and the results of the study may be hardly comparable with biomarker studies in which smaller A*β* aggregates or oligomers were investigated.

In 2007, Santos et al. established a method for A*β* oligomer detection based on fluorescence resonance energy transfer (FRET) and detection of FRET events by flow cytometry. To label oligomers in CSF, two fluorescence-labeled A*β* antibodies were added: 4G8-AlexaFluor 488 and 6E10-AlexaFluor 594. FRET means that energy is transferred from an excited donor (4G8-AlexaFluor 488) to an acceptor molecule (6E10-AlexaFluor 594) under defined spatial conditions, leading to fluorescence emission of the acceptor molecule. FRET events will only occur if both antibodies are in close spatial distance, that is, if both are bound to the same A*β* oligomer. A*β* monomers will not be detected as only one antibody probe can bind, respectively, and if two monomers are in close distance, the resulting FRET signal will be of very low intensity. The application of two specific A*β* antibodies in the detection process will ensure high specificity of the assay. The sensitivity of the method was investigated using synthetic ADDL and fibril preparations, determined to be linear in a range of 10 pM to 2.5 nM, and referred to the monomer concentration. The detection limit was set to be in the femtomolar range. Estimation of A*β* conformer size is possible due to analysis of the fluorescence intensities of each single particle. 174 human CSF samples of nondemented individuals, sample volume 200 *μ*L, were investigated in the assay. A large variation of the oligomer concentration was demonstrated, and a weak correlation between the age of the individuals and the oligomer concentration was stated. The assay was shown to be highly reproducible [46], but to date no data obtained from AD patient samples were published. Instead, in 2008, Santos et al. published a related method for the detection of A*β* oligomers in plasma samples. Simultaneously, the content of A*β*1-40 and A*β*1-42 could be quantified using species-specific A*β* antibodies for immunoprecipitation. The resulting immunocomplexes were immobilized to magnetic beads, and fluorescence-labeled antibodies were added. Subsequently, the samples were investigated by flow cytometry. The amount of A*β* oligomers allowed differentiation between 17 plasma samples of AD patients and 16 plasma samples of nondemeted controls with a specificity of 81.2% and a sensitivity of 70.6% [47].

In 2006, Birkmann et al. developed a method for the detection of single prion particles, counted by FCS. Prion aggregates in brain samples of BSE- and Scrapie-infected animals were isolated by chemical precipitation, labeled by two different specific and fluorescence-labeled prion antibodies, and detected in FCS using the two-color mode. As several probe antibodies bind to one aggregate, high fluorescence intensity peaks are detected if the aggregates cross the laser focus. Monomers can be distinguished by an intensity cut-off. Coincidental signals of both markers were counted as specific events. In 2007, the method was refined and used for the detection of prion protein aggregates in the CSF of BSE-infected cattle versus controls. In the new assay version, denominated Surface-FIDA (fluorescence intensity distribution analysis), the prion protein aggregates were immobilized to the surface of a glass chip using specific capture antibodies. Then, two fluorescence-labeled detection antibodies were applied for detection of the aggregates. A scheme of the measurement principle can be found in the figure. At least, three detection antibodies are used in the assay procedure, providing high specificity [48]. Additionally, detection of monomers can be excluded by the application of the same antibody as capture and as detection probe. In 2007, the assay was adapted to the detection of A*β* oligomers. The linearity of the assay was evaluated using synthetically prepared aggregated A*β*. The assay was shown to be linear over a wide range of A*β* aggregate amounts in the picogram range. Synthetic A*β* monomers were not detected. As a first trial to perform Surface-FIDA on real CSF samples, 20 *μ*L crude CSF of three AD patients and two nondemented control patients were subjected to the assay. The count of A*β* aggregates was higher in AD patients than in healthy controls. Recently, the assay could be optimized with regard to its biochemical steps and adapted to LSM, leading to further improvement of sensitivity. Additionally, using the image-based method, every single aggregate can be characterized with regard to its size and composition [49–51]. The influence of heterophilic antibodies, as described by Sehlin et al. [41], was not investigated yet but should be addressed in ongoing studies.

## 4. Conclusion

The interest of the Alzheimer's research community to detect A*β* oligomers and aggregates in body fluids grew strongly in recent years. A*β* oligomers have been shown to be the main toxic species in AD pathology, and the ability to quantify and qualify them could not only enhance AD diagnosis, but also help to investigate the contribution of A*β* oligomers and HMW particles to AD pathology. The development of techniques for the reliable detection and quantitation of aggregated A*β* species, however, is technically challenging, as already described in the introduction. 

As today indicated by six preliminary but independent studies, the concentration of A*β* oligomers was higher in CSF of AD patients than in healthy controls, and one study even reported on similar results in plasma samples [26–29, 43, 47, 49]. These results further strengthen the theory that A*β* oligomers might be a valuable marker for AD diagnosis and therapy monitoring. In contrast, Henkel et al. reported that there was no correlation between A*β* aggregate count and diagnosis [44]. These incoherent results can be explained by technological differences and differences in sample preparation. It was already suggested that standard operation procedures for sample preparation might lead to more consistent results, and several research networks are currently working on standardization of sample collection, preanalytical and analytical features for the harmonization of AD biomarker measurements. 

The finding that the concentration of A*β* oligomers is higher in CSF of AD patients than in healthy controls somehow seems to contradict ELISA studies suggesting that the total or monomer A*β* amount decreases with disease progression. Englund et al., however, found evidence that the lowering of A*β*42 might well be caused by its oligomerization [52]. Thus, the reported decrease of monomeric A*β*, which in fact might just be a decrease of accessible monomeric A*β*, might well be in good accordance with the observed increase of aggregated A*β* with disease progression. To obtain reliable information in the future hundreds of samples will have to be investigated employing different assay systems. Still, a lot of work is needed to elucidate the nature of the A*β* oligomers relevant to the disease as well as to improve the technical robustness of the applied quantification methods. Some of the assays reviewed here are complex and their robustness will have to be proven in future. After their technical optimization, the most robust and reliable assays could easily be adapted to other protein aggregates involved in the pathology of neurodegenerative diseases.

Although lumbar puncture is moderately invasive and has low incidence of complications [53], biomarkers detectable in blood plasma would be of great value for wider diagnostic use and for therapy monitoring. It would be very useful to adapt the most robust and sensitive methodology developed based on CSF to plasma and serum samples. Measurements in plasma, however, are technically much more challenging. A*β* is well known to bind to plasma proteins like albumin and lipoproteins, leading to masking of epitopes [54]. In 2009, Xia et al. reported on an A*β* oligomer specific ELISA. In this study, plasma levels of A*β*42 and oligomeric A*β* species were strongly correlated across the subjects [55].

In summary, several sensitive assays for the detection and characterization of A*β* oligomers in CSF are currently developed. Preliminary results indicate that A*β* oligomers and HMW particles might be valuable biomarkers for AD, and biomarker studies on aggregated A*β* species might enlighten the role of A*β* aggregates in progression of Alzheimer's disease.
